# Epigenetic treatment of solid tumours: a review of clinical trials

**DOI:** 10.1186/s13148-015-0157-2

**Published:** 2015-12-10

**Authors:** Clara Nervi, Elisabetta De Marinis, Giovanni Codacci-Pisanelli

**Affiliations:** Department of Medical and Surgical Sciences and Biotechnology, University of Rome “la Sapienza”, Corso della Repubblica, 97, 04100 Latina, Italy

**Keywords:** Epigenetic treatment, Histone deacetylases (HDACs), Histone methyltransferases (HMTs), DNA methylation, DNA-methyltransferases, ncRNAs, Azacytidine, Decitabine, Valproic acid, Suberoylanilide hydroxamic acid (SAHA)

## Abstract

Epigenetic treatment has been approved by regulatory agencies for haematological malignancies. The success observed in cutaneous lymphomas represents a proof of principle that similar results may be obtained in solid tumours. Several agents that interfere with DNA methylation-demethylation and histones acetylation/deacetylation have been studied, and some (such as azacytidine, decitabine, valproic acid and vorinostat) are already in clinical use.

The aim of this review is to provide a brief overview of the molecular events underlying the antitumour effects of epigenetic treatments and to summarise data available on clinical trials that tested the use of epigenetic agents against solid tumours. We not only list results but also try to indicate how the proper evaluation of this treatment might result in a better selection of effective agents and in a more rapid development.

We divided compounds in demethylating agents and HDAC inhibitors. For each class, we report the antitumour activity and the toxic side effects. When available, we describe plasma pharmacokinetics and pharmacodynamic evaluation in tumours and in surrogate tissues (generally white blood cells).

Epigenetic treatment is a reality in haematological malignancies and deserves adequate attention in solid tumours. A careful consideration of available clinical data however is required for faster drug development and possibly to re-evaluate some molecules that were perhaps discarded too early.

## Background

Research on tumour biology has provided conclusive evidence on the primary role of genetic alterations in the initiation and progression of cancer. However, the deregulation of epigenetic processes such as DNA methylation and alterations of “histone code” are equally important oncogenic factors *per se* [1–4]. Epigenetic processes affect the packaging of chromatin and direct distinct cellular gene expression programmes. They are heritable through cell division and do not involve changes in the DNA sequence [4–6]. Operating at the level of chromatin structure, epigenetic mechanisms play a key role during embryogenesis, X-chromosome silencing, cellular proliferation and differentiation and in disease states [2, 4–6]. They also facilitate a selective readout of the genome, thereby regulating stem cell developmental potential and cell fate. Subtle disturbances of the epigenetic framework in progenitor, differentiating or terminal cells may, besides well-known genetic alterations, promote carcinogenesis [7, 8]. The dynamic and reversible nature of epigenetic mechanisms makes these processes of therapeutic relevance in many diseases including cancer.

**Fig. 1:**
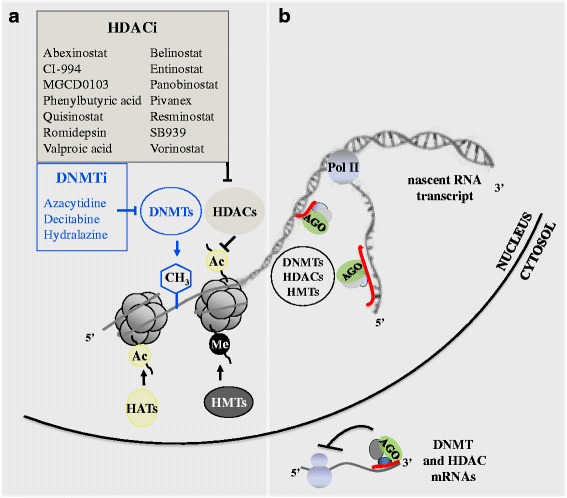
Schematic representation of gene expression regulation by epigenetic drugs, components of the DNA and chromatin-modifying machinery and ncRNAs. **a** Epigenetic drugs reported to be effective against cancer cells inhibit the activity of DNA methyltransferases (DNMTi) or histone deacetylases (HDACi). DNMTs add a methyl group (CH_3_) to the 5′ carbon atom of cytosine in DNA CpG dinucleotides. DNMTs also participate in multiprotein chromatin-modifying complexes containing histone deacetylases (HDACs) and histone methyltransferases (HMTs),which induce post-translational modifications of lysine residues in the amino terminal tails of nucleosomal histones, including deacetylation (HDACs), methylation (HMTs) and acetylation (histone acetyltransferases (HAT). Specific molecular modifications on CpGs and nucleosomal histones affect the higher order of chromatin architecture and function by changing the interaction of histones with DNA or the contact between different histones in adjacent nucleosomes. This allows or denies the accessibility of the transcriptional machinery and DNA-binding proteins to specific sites on genome, resulting in activation or silencing of gene transcription. *Ac* acetylation, *Me* methylation. **b** Short and long ncRNA are emerging as novel regulators of chromatin structure, alternative to DNA-binding proteins. They can act as key specificity determinants for epigenetic regulation of gene expression. In the nucleus, both short and long ncRNAs can bind complementary sequences on DNA or nascent RNA transcripts and guide the Argonaute-containing complexes (Ago) to recruit HDACs, HMTs and DNMTs for gene silencing. Nascent lncRNAs can also be tethered to the locus from which they are transcribed through association with RNA polymerase II (Pol II). In the cytosol, microRNAs and siRNAs act as post-transcriptional regulators of the expression of HDAC and DNMTs through their complementarity with mRNA sequences

Epigenetic processes involve methylation of DNA and post-translational modification of nucleosomal histones, which contribute to a complex “epigenetic code” that superposes the nucleotide sequence to direct gene expression [4, 9–11] (Fig. 1a).

DNA methylation results from the transfer of a methyl group from a methyl donor substrate, namely S-adenosyl-L-methionine (AdoMet), to the 5′ position of a cytosine in a CpG context. CpG dinucleotides can be sparse or tend to be gathered in repetitive sequences in or around gene promoters in regions known as CpG islands. The methylation status of CpG moieties within regulatory DNA sequences affects the transcription of the related gene [2, 10]. The creation of DNA methylation patterns during the embryogenesis establishes the compartmentalization of genome into transcriptionally active and inactive domains. DNA methylation is fundamental for a correct expression of imprinted genes, chromosomal dosage compensation (X-chromosome inactivation) and tissue-specific gene expression [2, 3, 12–14].

The oncogenic effect of DNA methylation is mainly related to the formation of a repressive chromatin structure on promoter regions that impairs the constitutive expression of genes involved in cell cycle regulation, DNA repair, apoptosis, differentiation, drug resistance, angiogenesis and metastasis [1–4]. A family of enzymes known as DNA methyltransferases (DNMTs) catalyses the DNA methylation reaction. DNMT1 is a maintenance methylase that recognises and methylates hemi-methylated CpG dinucleotides during DNA replication allowing the propagation and conservation of the DNA methylation patterns through the future generations [14, 15]. DNMT3a and 3b generally act as *de novo* methylases. They are highly expressed in embryonic stem cells, early embryos and developing germ cells and, at a low rate, in somatic tissues or postnatal animals. DNA methyltransferase-3-like (DNMT3-Like) lacks enzymatic activity but may be essential for the establishment of maternal methylation imprints and the appropriate expression of maternally imprinted genes. The inhibitory effect of CpG island methylation on gene expression is mediated by the involvement of proteins with high affinity for methylated CpGs. These methyl CpG-binding proteins (MeCP1, MeCP2, MBDs and Kaiso) [16–21] exert their function as transcriptional repressors via chromatin modification. Methyl CpG-binding proteins are often part of large repressor complexes as NuRD, NoRC, mSin3A and SWI-SNF. Repressor complexes recruit histone deacetylases (HDACs) and histone methyltransferases (HMTs) on methylated target promoter sequences. These enzymes catalyse covalent post-translational modifications of specific residues on histone 3 (H3) and 4 (H4) N-terminal tails (e.g. deacetylation of lysine (K) 9, demethylation of K4 and methylation of K9 and K27 of H3), inducing a compacted transcriptionally inactive chromatin structure. Histone acetylation status also depends on the contrasting activities of HDACs and histone acetyltransferases (HAT) group of enzymes. The latter are presumed to induce histone tail modifications (e.g. acetylation of K9 and K14 of H3), resulting in a transcriptionally active chromatin state. As for histone acetylation, histone lysine methylation can be dynamically regulated by the recruitment of members of the histone lysine methyltransferases and demethylase class of enzymes, which impose memory on gene transcription [22]. Other histone tail modifications include phosphorylation, sumoylation, ubiquitination and ADP ribosylation. Overall, DNA methylation and histone modifications work together to assemble a chromatin structure, which dynamically shifts from a transcriptionally permissive state to a transcriptionally inactive state and vice versa [2, 14].

Inhibition of HDACs can be achieved in normal conditions by endogenous molecules [23] explaining the plausibility of this process in the normal regulation of gene expression.

Aberrant DNA methylation and chromatin modifications, altering gene transcription states, are common hallmarks of human tumour cells [24]. Studies on leukaemias have provided paradigmatic examples for the functional implications of genetic and epigenetic alterations in cancer development [25, 26]. These studies underline the possibility of reversing disease-associated aberrant epigenetic states by targeting the catalytic activities of chromatin remodelling enzymes. Thus, these enzymes are attractive targets for therapeutic intervention in cancer [27–29]. The possibility of drug development in this field has recently been reviewed [22, 24, 30, 31].

Evidence is growing that non-coding RNAs (ncRNAs) are involved in inducing chromatin modifications and act as additional molecular determinants for epigenetic regulation of gene expression also in human cells [32–34] (Fig. 1b). NcRNAs comprise a large and heterogeneous family of RNA molecules differing in length (short, such as microRNAs, and long ncRNAs), which are transcribed from DNA but not translated into proteins. By regulating gene expression at the transcriptional and post-transcriptional level, they affect a broad range of physiologic functions and pathologies such as neoplastic diseases [35, 36]. Both short and long ncRNAs appear to function by guiding the recruitment HDACs, HMTs and DNMTs, and other proteins involved in the epigenetic regulation of transcription, to homology-containing loci on gene promoters and in the genome. Moreover, short ncRNA, as microRNAs and siRNAs, can repress the expression of HDAC, DNMTs and other components of chromatin-modifying complexes at the post-transcriptional level by interacting with their mRNAs [32, 33, 35] (Fig. 1b). Therefore, ncRNAs play direct roles in DNA methylation, heterochromatin formation, histone modification and gene silencing. In turn, they are epigenetically targeted for repression or activation; this can be a valuable way of amplifying changes in the levels of downstream effectors. Knowledge of these emerging regulatory roles of ncRNAs has implication not only in cellular physiology and pathology but also for the development of novel epigenetic drugs that re-establish the correct pattern of gene expression in complex diseases such as cancer.

**Fig. 2:**
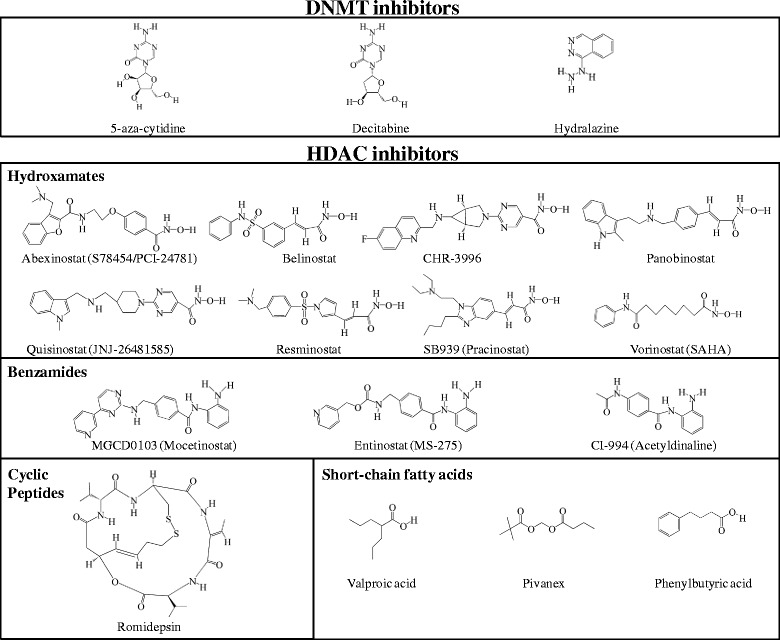
Chemical structures of different classes of DNMT and HDAC inhibitors. Antimetabolites 5-azacytidine and decitabine (5-aza-2′-deoxycytidine) are cytidine analogues; these nucleoside derivatives are incorporated into DNA leading to covalent adduct formation, thus acting as mechanistic inhibitors. The non-nucleoside DNMT inhibitor hydralazine interacts within the binding pocket of the enzyme interfering with the DNA methylation mechanism

Currently in the clinical setting, there are two classes of epigenetic drugs, which act through the inhibition of the enzymatic activities responsible for epigenetic transcriptional silencing: DNMTs and HDACs (Fig. 2). DNA methylation inhibitors 5-azacytidine (azacytidine) and 5-aza-2′-deoxycytidine (decitabine) have been approved by the US Food and Drug Administration (FDA) in 2004 and 2006, respectively, for the treatment of patients with myelodysplastic syndromes (MDS). US FDA approved the HDAC inhibitors suberoylanilide hydroxamic acid (SAHA, vorinostat, in 2006) and romidepsin (depsipeptide, in 2009) for the treatment of patients with progressive, persistent or recurrent cutaneous T-cell lymphoma [37]. In 2015, FDA approved panobinostat in combination with bortezomib and dexamethasone for the treatment of patients with multiple myeloma [38] and belinostat for the treatment of patients with peripheral T-cell lymphoma (PTCL) [39].

The success of epigenetic therapies in inducing clinical responses in MDS and lymphoma not only gave to this kind of treatment high visibility, but it also suggested that similar results might be obtained in solid tumours and that this line of research deserves proper evaluation. The relevance of epigenetic treatment in haematological malignancies (leukaemias, lymphomas, myelodysplastic syndromes, myeloma) have already been described in detail [40].

In clinical studies, epigenetic treatment was administered alone or in combination with standard anticancer therapies (usually chemotherapy, sometimes radiotherapy) to improve their antitumour activities [41]. In some cases, the aim was more directly related to the control of gene activity and to prevent the development of resistance due to the overexpression of a specific gene, for example thymidylate synthase [42].

In several papers [43, 44], particularly those more recently published [45, 46], a detailed explanation for the combination of epigenetic and standard anticancer treatment is provided together with complete and stimulating results.

The present review will focus on the epigenetic treatment of solid tumours*:* we collected data from clinical studies available as full papers, data on ongoing studies are available at clinicaltrials.gov site and have been reviewed [47].

In the text, we discuss data on the pharmacology, pharmacokinetics (PK) and pharmacodynamics (PD), on the toxic side effects and on the antitumour activity of epigenetic treatments. In the tables, we give more details for epigenetic treatment alone (Table 1) or in combination with traditional anticancer agents or radiotherapy (Table 2).

**Table 1:** Studies on epigenetic treatment alone

Drug (s) and schedule	Study type - histology (number of patients)	Results and data provided	Reference
Abexinostat (S78454/PCI-24781) p.o. 60 mg/m2 bid 4 days on/3 days off	Mixed tumours (15 pts)	PK/PD model predicts thrombocytopenia	[90]
Azacytidine + Valproate Aza s.c. for 10 days q. 28 days. MTD 75 mg/m^2^/day VPA in plasma 75–100 μg/ml ^(a)^	Dose escalation Mixed tumours (55 pts)	PBMC: DNA methylation decreased. H3 acetylation increased. Patients with stable disease had more H3 acetylation. DLT: neutropenic fever and thrombocytopenia	[68]
Azacytidine +Entinostat Aza 30–40 mg/m2/day for days 1–6 and 8–10 q 28 days Entinostat 7 mg/m2 days 3 and 10	Phase I/II trial NSCLC (45 pts)	Demethylation of 4 epigenetically silenced genes (CDK2a, CDH13, APC, RASSF1a). In plasma DNA was associated with improved progression-free and overall survival	[62]
Azacytidine (AC) Phenylbutyrate (PHB) AC 10-25mg/m2/day for 21 days, 75 mg/m2/day for 7 days PHB 200–400 mg/m2 days 6, 13 and 20	Phase I Mixed tumours (27 pts)	Toxicity: neutropenia, anaemia. No PK interaction “No conclusive statement can be made on histone acetylation or methyltrasferase activity”.	[91]
Decitabine + VPA Dec 5–15 mg/m2/day for 10 days. VPA 10–20 mg/kg/day for days 5–21 q 28 days	Phase I NSCLC (8 pts)	Neurological toxicity. Increase in foetal Hb levels in all pts	[63]
Belinostat 1000 mg/m2/day i.v. for days 1–5 q 21 days	Phase II Ovarian: platinum resistant (18 pts) or micropapillary (14 pts)	Toxicity: thrombosis (3 pts). Increased H3 acetylation in PBMC and in two tumours	[73]
Belinostat 1000 mg/m2/day i.v. for days 1–5 q 21 days	Phase II Refractory Thymic epithelial tumours (41 pts)	Nausea, vomiting, fatigue Modest activity Protein acetylation did not predict outcome	[92]
Belinostat 600–1400 mg/m2/day i.v. for days 1–5 q 3 weeks .	Phase I/II Hepatocarcinoma (60 pts)	PK linear. MTD not reached at 1400 mg/m2. Toxicity: abdominal pain, liver toxicity, vomiting. Plasma concentrations higher than effective *in vitro* levels for 4 hours. Disease stabilisation. High HR23B associated with more stabilisation.	[93]
Belinostat 1000 mg/m2/day i.v. for days 1–5 q 21 days	Mesothelioma (pre-treated) (13 pts)	Not active in terms of RR toxicitiy: nausea, emesis, fatigue and constipation. One fatal cardiac arrhythmia	[94]
Belinostat, (oral formulation) 150–1000 mg/m2/day for days 1–5 q 21 days	Pharmacological evaluation Mixed tumours (46 pts)	PK and PD: results similar to the parenteral formulation	[95]
CHR-3996 5–160 mg/day p. o. RD 40 mg/day p.o.	Phase I Mixed tumours (39 pts)	DLT: thrombocytopenia, fatigue, atrial fibrillation, ECG alterations, elevated creatinine. AUC proportional to dose, plasma concentration sufficient for preclinical antitumour activity Effect on histone acetylation in PBMC	[45]
CI-994 2–8 mg/m2/day RD 8 mg/m2/day for 8 weeks q 10 weeks	Phase I Mixed tumours (53 pts)	Toxicity: Thrombocytopenia (DLT). PK data.	[48]
MGCD0103 12.5-56 mg/m2/day p.o. 3 times/week for 2 weeks q 3 weeks RD 45 mg/m2/day	Phase I Mixed tumours	Inhibition of HDAC activity and induction of acetylation of H3 histones in peripheral WBCs	[96]
MS-275 MTD 10 mg/m2 q14 days	Phase I Mixed tumours (31 pts)	Toxicities: nausea, vomiting, anorexia, fatigue. Half-life 39–80 hrs (longer than expected). Linear PK. Increased H3 acetylation in PBMC. Peak plasma levels higher than effective *in vitro* concentration.	[97]
Panobinostat 20 mg p.o. twice/week	Pharmacological study Mixed tumours (36 pts)	No effect of food on PK parameters	[98]
Panobinostat 40 mg p.o. three times/week	Sarcoma (47 pts) Ovarian Sex Cord Tumours (5 pts)	Poorly tolerated. No activity in sarcoma. Activity in OSCT Toxicity: thrombocytopenia, fatigue, anaemia	[99]
Panobinostat 20 mg/m2 for days 1 and 8 q 21 days	Prostate (35 pts)	No clinical activity Toxicity: fatigue, thrombocytopenia, nausea	[100]
Panobinostat 20 mg	Mixed tumours (4 pts)	PK determined by trace radiolabelled 14C excretion Rapid oral absorption, liver and renal excretion	[101]
Pivanex 2.34 g/m2/day in 6 h for 3 days q 21 days	Phase II NSCLC (47 pts)	Toxicity: fatigue, nausea, dysgeusia- 3 partial responses (6%)	[102]
Quisinostat (JNJ-26481585)	Mixed tumours (92 pts) Phase I RD 12 mg days 1,3 and 5	Toxicity: cardiovascular, fatigue, nausea PD: increased H3Ac in hair follicles, skin and tumour.	[103]
Resminostat RD 600 mg/day p.o. for 5 days q 14 days	Phase I Head-and-neck refractory	Toxicity: nausea, vomiting, fatigue. PK data, HDAC inhibition, H4Ac increase in PBMC	[69]
Romidepsin 13 mg/m2 i.v. in 4 h for days 1, 8 and 15 q 28 days	Phase II Refractory Prostate (35 pts)	Toxicity: nausea, fatigue, vomiting and anorexia No antitumour activity	[104]
Romidepsin 13 mg/m2 in 4 h, for days 1, 8 and 15 q 28 days	Phase II Head and Neck (14 pts)	Toxicity: nausea, vomiting, constipation, fatigue H3 hyperacetylation in PBMC Reduced or stable Ki67 On microarray 641 differentially expressed genes No consistent change ion methylation of specific genes Upregulation of p21Waf1/Cip1.	[70]
Romidepsin New schedule: 1–9 mg/m2 in 4 h for days 1, 3, and 5 q 21 days RD 7 mg/m2	Phase I Mixed tumours (28 pts)	Increase in 3HAc in PBMC. PK data described. Toxicity: ECG changes	[54]
SAHA 400 mg/day p.o.	Phase II Head-and-neck, refractory (13 pts)	No response. Toxicity: anaemia, anorexia, hyperglycemia, thrombocytopenia, dehydration	[82]
SAHA 400 mg/day p.o.	Phase II Refractory Prostate (27 pts)	IL-6 was higher in patients with toxicity (Fatigue, nausea)	[105]
SAHA 400 mg for 14 days q 21 days	Phase II Glioblastoma (66 pts)	Analysis of tumour tissue. Increased Acetylation of H2A, H3, H4. up-regulation of e-regulin. PK influenced by enzyme-inducing drugs. Toxicity: fatigue, thrombocytopenia. nausea, diarrhoea	[75]
SAHA 400 mg for 14 days q 21 days	Phase II Breast (14 pts)	No antitumour activity. Toxicity: Fatigue, nausea, diarrhoea, and lymphopenia	[106]
SAHA 400 mg for 14 days q 21 days	Phase II Ovarian (27 pts)	No antitumour activity Toxicity: Neutropenia, Leukopenia, Thrombocytopenia, Constitutional, Gastrointestinal, Metabolic	[107]
SAHA 400 mg for 14 days q 21 days	Phase II NSCLC second line (16 pts)	No antitumour activity Toxicity: fatigue, dehydration, hyperglycemia, mild myelosuppression	[108]
SAHA 400–800 mg for 14 days q 21 days RD 400 mg for 14 days q 21 days	Breast, colorectal, NSCLC (16 pts)	No antitumour activity. Toxicity anorexia, asthenia, nausea, thrombocytopenia, vomiting, weight loss	[60]
SAHA 400 mg for 14 days q 21 days	Thyroid (19 pts)	No antitumour activity Toxicity: fatigue, dehydration, ataxia, pneumonia, bruises, thrombosis, thrombocytopenia	[109]
SAHA 600 mg bid days 1–3 q 7 days or 400 mg for 14 days q 21days	Phase I Gastrointestinal (16 pts)	DLT thrombocytopenia. Some PK data: AUC μM/h 7.75±2.79 for 400 mg; 3.94±1.56 with 300 mg. t ½ 1.05±0.32 – 1.49±0.82 hours	[110]
SAHA 100–500 mg once or twice daily for 14 days q 21 days	Phase I Mixed tumours (18 pts)	MTD not reached. Recommended dose 500 for once, 200 for twice daily. Some PK data: AUC linear with dose	[111]
SAHA 300 or 400 mg bid days 1–3 q 7 days	Mesothelioma (pretreated) (13 pts)	2 PR. Toxicity: fatigue, anorexia, dehydration, diarrhea, nausea, and vomiting	[112]
SAHA 2 h i.v. infusion 75–900 mg/m2/day days 1–3 q 21 days or 300–900 mg/m2/day days 1–5 q 21 days	Phase I Mixed tumours (37 pts)	Toxicity: myelotoxicity, fatigue, anorexia, hyperglicemia Increase in acetylated histones in PBMC and in tumour cells. PK data.	[113]
SAHA oral MTD 400 mg/day or 600 mg/day days 1–3 q 7 days	Phase I Mixed tumours (73 pts)	Toxicity: anorexia, dehydration, diarrhea, and fatigue. In PBMC acetylation increased 2 hrs after dose, back to basal levels at 8 hours	[114]
SAHA 300 mg tid	Breast (25 pts)	Decrease of proliferation-associated genes. No effect on methylation	[115]
SAHA 400 mg daily continuously	Melanoma (39 pts)	Toxicity fatigue, nausea, lymphopenia, and hyperglycemia. Some biochemical correlative data presented.	[116]
SAHA 300 mg tid days 1–3, 8–10, 15–17 q 21 days	Mesothelioma (pretreated) (329 pts)	Randomised phase III: no benefit Toxicity: fatigue or malaise	[117]
SB939 10–80 mg/day p. o. 3 times/week for 3 weeks q 4 weeks RD 60 mg/day	Phase I Mixed tumours (30 pts)	DLT: fatigue, hypokalemia, ECG alterations. AUC proportional to dose. HDAC increases at doses 60 mg.	[118]
SB939 10–90 mg daily five times a week q 2 wks RD 60 mg/day	Phase I Mixed tumours (38 pts)	PK data. No correlation of AcH3 and response. Toxicity: fatigue, nausea, vomiting.	[83]
Valproate intravenous infusion in 1 h 30-250 mg/kg/day for days 1–5 q 21 days RD 60 mg/kg/day	Phase I Mixed tumours (26 pts)	Toxicity: neurological. HDAC2 decreased; H3 Acetylation increased; VPA plasma levels 0.3-0.9 mM.	[80]
Valproate p. o. 20-40mg/kg/day for 5 days	Phase I Cervical cancer (12 pts)	VPA in plasma 73–170 μg/ml. (0.4-1 mM) No correlation of H3 acetylation in tumour biopsies and plasma VPA. Toxicity: Depressed consciousness	[79]
Valproate 500 mg p. o. tid (target concentration 50–100 μg/ml) (0.3-0.6 mM)	Phase II Low-grade Neuroendocrine (8 pts)	Two tumours had a 2-4-fold increase in Notch-1 mRNA, 3 had a decrease.	[71]

The references are included at the end of the text

*5FU* 5-Fluorouracil, *5mC* 5-methyl Cytosine, *AUC* area under the curve (also a dosing calculation for Carboplatin), *Bid* bis in die (twice a day), *DLT* dose-limiting toxicity, *FEC* combination of Fluorouracil, Epirubicin, Cyclophosphamide, *FolFOx* combination chemotherapy of Folinic acid, 5-Fluorouracil and Oxaliplatin, *GI* gastrointestinal, *i.v.* intravenously, *MTD* maximum tolerated dose, *NSCLC* non-small cell lung cancer, *PBMC* peripheral blood mononuclear cells, *PD* pharmacodynamic, *PFS* progression-free survival, *PK* pharmacokinetics, *p.o.* per os (orally), *PR* partial response, *Pt* patient, *q* every (Latin “quaque”), *RA* rapid acetylator (Hydralazyne metabolism), *RD* recommended dose, *RR* response rate, *SA* slow acetylator (Hydralazyne metabolism), *SAHA* Vorinostat, Zolinza ®, *TS* thymidylate Synthetase, target enzyme for 5FU activity, *VPA* Valproic Acid, *WBC* white blood cells

(1) Oral dose of VPA titrated in each patient to obtain adequate plasma concentrations.

**Table 2:** Epigenetic treatment associated with a conventional anticancer agent

Epigenetic drug	Tumour type and chemo	Results and data provided	Reference
5-azacytidine RD 75 mg/m2/day days 1–4 and 15–18 q 28 days	Phase I Erlotinib 150 mg/day Mixed tumours (30 pts)	Aza: increasing dose (75–100) and days of treatment (2–8) Toxicity: rash, diarrhoea, nausea, and fatigue	[119]
5-azacytidine	Ovarian cancer, platinum insensitive (30 pts)	Toxicity: fatigue, myelosuppression DR4 methylation in PBMC related to activity	[120]
5-azacytidine 75 mg/m2/day for days 1-5	Prostate cancer (22 pts) Docetaxel (day6) , prednisone	Toxicity: myelosuppression Reduction in GADD-45 methylation (peripheral DNA) on day 5 Only pts that had a reduction had a response.	[61]
Abexinostat 15–45 mg tid days 1-5	Sarcoma 22 pts Doxorubicin 75 mg/m2 day 4	Neutropenia (growth factors required), fatigue, thrombocytopenia, and anemia. PK of Abexinostat described. HDAC levels inhibited in PBMC	[121]
Belinostat 1000 mg/m2/day for 5 days	Carboplatin AUC 5 day 3 Resistant ovarian cancer (29 pts)	Toxicity: neutropenia, thrombocytopenia, vomiting No effect, study closed	[122]
Belinostat 1000 mg/m2, 48 h c.i.	Thymic epithelial (26 pts) Cisplatin, doxorubicin, cyclophosphamide	Toxicity: nausea, diarrhea, neutropenia, thrombocytopenia, Immunomodulatory effect observed	[123]
Belinostat 1000 mg/m2 i.v. for days 1–3 then p.o. 2000 mg for days 4-5	Unknown primary (44 pts) Paclitaxel, carboplatin	Randomised phase II. No clinical benefit	[124]
CI-994 6 mg/m2/day for days 1–21, 28-day cycle	Phase II Pancreas. Gemcitabine 1000 mg/m2 days 1, 8 and 15 (174 pts)	Increased incidence of neutropenia and thrombocytopenia No improvement of gemcitabine activity	[125]
CI-994 4–10 mg/m2/day RD 6 mg/m2/day for days 1–14 q 21 days	Phase I Mixed tumours (54pts) Capecitabine 1650–2000 mg/m2/day for 14 days q 21 days	PK not altered by capecitabine. Toxicity: Thrombocytopenia	[49]
Decitabine 45–135 mg/m2 6 h infusion for day 1 RD 90mg/m2	Phase I Carboplatin (AUC 5 or 6) day 8 Mixed tumours (33 pts)	Dose dependent, reversible demethylation in PBMC maximally at day 10. Demethylation of the MAGE1A gene Toxicity: myelosuppression	[42]
Decitabine 10 mg/m2/day for 5 days	Carboplatin AUC 5 day 8 Ovarian cancer (17 pts)	35% RR 10.2 months PFS In PBMC and tumours global and gene-specific demethylation. Demethylation of MLH1, RASSF1A, HOXA10, HOXA11 correlated with PFS	[74]
Decitabine 0.15 mg/kg i.v. daily × 5 days/wk for 2 wks	Phase I-II Temozolomide p.o. 75 mg/m2 daily for weeks 2–5 of a 6-week cycle Refractory Melanoma (35 pts)	Toxicity: mainly haematological No effect on promoters of DNA repair genes Excellent PK and PD data (also in tumours)	[55]
Decitabine days 1–5 q 28 days 10–20 mg/m2/day	Phase I Ovarian, recurrent (10 pts) Carboplatin AUC 5 day 5	Toxicity: myelosuppression, nausea, fatigue global and gene-specific DNA methylation	[126]
Decitabine i.v. day 1 45 mg/m2	Carboplatin AUC 6 day 8 Ovarian cancer (15 pts)	Patients with methylated hMLH1 tumour DNA in plasma Decitabine appears to reduce the efficacy of carboplatino Decrease in global levels of methylation with Decitabine.	[127]
Decitabine 01–0.2 mg/kg 3 days weekly Panobinostat 10-30mg q 4 days	Temozolomide 150–200 mg/m2/day Resistant melanoma	Toxicity: myelosuppression, fatigue, nausea No antitumour acitivity.	[128]
Entinostat 10 mg p.o. day 1 and 15 q 28 days	Randomised phase II NSCLC (132 pts) Erlotinib 150 mg/day	Toxicity: rash, fatigue, diarrhoea, nausea High E-caderin levels associated with longer PFS	[86]
Entinostat 1–5 mg/kg days 1,8,15 q 28 days	Phase I Mixed tumours (19pts) 13-cis retinoic acid 1 mg/kg	Toxicity: hyponatremia, neutropenia, anaemia. PD: Increased histone acetylation	[129]
Entinostat 5 mg/week	Breast (64pts) Examestane 25 mg/day	Randomised phase II. Patients had progressed with AI. Protein lysine hyperacetylation associated with prolonged PFS	[130]
Entinostat 10 mg/2 weeks	Phase I Mixed tumours (31 pts) Sorafenib (400 mg tid)	Toxicity: Handfoot syndrome, nausea/vomiting, and fatigue	[131]
Hydralazine (182 mg RA; 83 mg SA) + Valproate (40 mg)	Phase II (17 pts) Mixed tumours: re-treatment of resistant patients with same chemo as before	Toxicity: mainly haematological Reduction in global DNA methylation, histone deacetylase activity, and promoter demethylation	[58]
Hydralazine (182 mg RA; 83 mg SA) + Valproate (30 mg)	Phase II Progressive Cervical cancer (36 pts) Cisplatin + Topotecan	Advantage in PFS (10 vs. 6 months) Molecular correlates pending.	[59]
Hydralazine (182 mg RA; 83 mg SA) + Valproate (30 mg)	Phase II Breast (16 pts) Doxorubicin, cyclophosphamide	Decrease in 5mC content and HDAC activity. Up- and down-regulation of many genes.	[57]
Panobinostat alone: 20 mg for days 1,3 and 5 for 2 weeks q 3weeks with chemo: 15 mg	Prostate (pretreated) (16 pts) Docetaxel 75 mg/m2 q 21 days	Toxicity: dyspnea, neutropenia Increase in histone acetylation in PBMC No relevant antitumour actitvity	[132]
Panobinostat 30 mg/day, days 1,3 and 5 q 14 days	Recurrent glioma (12 pts) Bevacizumab 10 mg/kg q 14 days	Toxicity: thrombocytopenia, hypophosphatemia, hemorrhage, thrombosis.	[133]
Panobinostat 10 mg days 1,3 and 5 q 14 days	Phase I Mixed tumours (12 pts) Bevacizumab 10 mg/kg q 14 days Everolimus 5 or 10 mg	Toxicity: Mucositis, arrhythmia. No consistent change in HDAC activity in PBMC	[134]
Panobinostat 30 mg days 1 and 4	NSCLC, HNC Erlotinib 100 mg/day	DLT: cardiac, nausea. Fatigue. PK and PD data.	[135]
Panobinostat 20 mg	Gleevec-resistant GIST (12 pts)	No actibvity but evidence of 3HAc increase in PBMC	[136]
Panobinostat 10 mg days 1, 3 and 5	Paclitaxel, Carbopaltin AUC=5 Phase I Miscellaneous tumours (12 pts)	Toxicity: diarrhea, fatigue, and vomiting	[137]
SAHA 100-400mg/day RD 300 mg	GI carcinoma (14 pts) Radiotherapy 30 Gy in 3 Gy/day over 2 weeks	Toxicity: fatigue Diarrhoea proportional to the volume of intestine irradiated.	[41, 138]
SAHA 200–800 mg/day 1week q 2 weeks RD 600 mg/day	Refractory colorectal FolFOx (21 pts)	Toxicity: fatigue, anorexia, dehydration no consistent modulation of TS expression	[88]
SAHA days 1–3 q 14 days 600–2000 mg/day RD 1700 mg once 600 mg tid	Phase I Refractory colorectal (43 pts) FU-LV	toxicity: neutropenia, thrombocytopenia, fatigue, nausea or vomiting, anorexia, mucositis. No consistent effect on biopsies.	[139]
SAHA 400 mg/day	Tamoxifen (43 pts) Hormone-resistant breast	Histone hyperacetylation and higher baseline HDAC2 levels that correlated with response	[84]
SAHA RD 400 mg/day 14 days q 21 days 600 mg/day bid q 21days	Phase I (28 pts) Mixed tumours Paclitaxel (200 mg/m2) (Carboplatin (AUC 6)	Toxicity: emesis, neutropaenia, fatigue	[140]
SAHA 400 mg/day	Randomised Phase II vs. placebo NSCLC 94 pts Paclitaxel Carboplatin	Toxicity thrombocytopenia, nausea, emesis, fatigue. RR 34% vs. 12%	[85]
SAHA 400 mg/day	Phase I-II resistant colorectal 5FU-leucovorin	Failed to establish an MTD Toxicity: fatigue, thrombocytopenia and mucositis. Intratumoral TS downregulation in one patient. Acetylation of H3 in PBMCs	[141]
SAHA 200–300 mg bid days 1–3 q 7 days	Phase I-II Breast (54 untreated pts) Paclitaxel 90 mg/m2/week Bevacizumab 10 mg/kg	Increased diarrhoea with the addition of SAHA Increased acetylation of Hsp90 and α-tubulin	[66]
SAHA 400 mg/day 14 days	Phase II Glioblastoma (37 pts) Bortezomib 1.3 mg/m2/day days 1,4,8 and 11 q 21days	Toxicity: Fatigue. No therapeutic advantage	[142]
SAHA 100–200 mg/day for days 1–14 q 21 days	Phase I Mixed tumours 12 patients Docetaxel 50–75 mg/m2 day 4 q 21 days	Excessive toxicity: neutropenic fever, cardiac, bleeding No PK interaction	[143]
SAHA + VPA for days 1–2 400–100 mg/day RD 800 mg/day	Phase I (32 pts) Mixed tumours Doxorubicin on day 3 20 mg/m2 weekly	Toxicity: fatigue, nausea HDAC2 expression in PBMC similar to tumours no correlation of SAHA levels with acetylation	[144]
SAHA 300 mg/day for 16 days q 28 days	Phase I Mixed tumours (22pts) Marizomib 0.15-0.7 mg/m2 i.v. days 1, 8 and 15 q 28 days	Toxicity: Fatigue, nausea, diarrhea, vomiting, PK data. Data on proteasome inhibition in PBMC	[76]
SAHA 300 mg for days 1–3 q 8 days	Bortezomib 1.3 mg/m2 days1,8 and 15 q 21 days NSCLC (21 pts)	Preoperative treatment. Toxicity: fatigue and hypophosphatemia	[145]
SAHA 200–300 mg tid for days 1–4 and 8-11	Bortezomib 1–1.3 mg/m2 for day 9 Phase I (60 pts)	Comparison in PBMC and biopsies after SAHA and SAHA-Bort. Dcreased Nur77 expression.	[146]
SAHA 400 mg p.o. for days 1–7 and 15–21 q 28 days	NSCLC (33 pts) Erlotinib-resistant Erlotinib 150 mg/day	No clinical activity Toxicity: anemia, fatigue and diarrhoea.	[147]
SAHA 300–400 mg/day for days 1-14	Gastric (30 pts) Capecitabine, Cisplatin	Toxicity: thrombocytopenia, fatigue, stomatitis, anorexia H3Ac correlated with SAHA dose	[148]
SAHA 200–400 mg p.o. for days 1–14 q 21 days	Mixed tumours (35 pts) Sorafenib 400 mg p.o. bid	Recommended dose for SAHA 300 mg/die, but not tolerated. Toxicity: hand-foot syndrome. No tumour response.	[149]
SAHA tid for days 1–4 and 8–11 q 21 days	Mixed tumours (29 pts) Bortezomib 1.3 mg/m2 i.v. for days 1, 4, 8 and 11	Toxicity thrombocytopenia, fatigue, increased ALT, elevated INR, and diarrhea. PK data provided.	[150]
SAHA p.o. for days 1–14 MTD 400 mg	Mixed tumours (23 pts) Bortezomib i.v. for days 1, 4, 8 and 11 q 21 days. MTD 1.3 mg/m2	Toxicity: fatigue, hyponatremia, nausea, anorexia Some PK data	[151]
SAHA 300 mg daily	Mixed tumours (78 pts) Pazopanib 600 mg daily	Toxicity: thrombocytopenia, neutropenia, fatigue, hypertension, diarrhea, vomiting	[152]
SAHA 400 mg daily	Gefitinib 250 mg NSCLC pretreated (52 pts)	No clinical benefit Toxicity: anorexia, diarrhea, fatigue, anemia	[153]
Valproate 30–90 mg/kg/day for days 1–5 q 21 days MTD 75 mg/kg/day	Karenitecin i.v. 0.8-1 mg/m2/day for days 3–7 q 21 days Melanoma: xenografts Phase I-II (39 pts)	Toxicity: somnolence, fatigue VPA levels at MTD 1.28 mMol Histone hyperacetylation was observed in PBMC. No effect of valproate on Karenitecin PK	[154]
Valproate 15–160 mg/kg/day for days 1–3 RD 120 mg/kg/day	Phase I (44 pts) Mostly breast FEC day 3	Toxicity: somnolence, myelosuppression Histone acetylation in tumour samples and in PBMCs correlated with valproic acid levels and was further linked to baseline HDAC2 but not to HDAC6 expression	[43]
Valproate 10–90 mg/kg/day	Melanoma (32 pts) Dacarbazine 800 mg/m2 q 21 days, interferon-α 600.000 IU twice daily	Toxicity: neurological, myelosuppression Acetylation in PBMC measured. “casting some doubts on the clinical use of VPA in this setting”.	[155]
Valproate Dose escalated to obtain active plasma concentration	Mesothelioma resistant to cisplatin (45 pts) Doxorubicin 60 mg/m2 q 21 days	Toxicity: myelosuppression 16% partial response rate	[156]
Valproate 15–160 mg/kg/day for days 1–3 MTD 140	Phase I Mixed tumours (48pts) Epirubicin 100 mg/m2 for day 3	Toxicity: somnolence, confusion, neutropenia VPA levels correlate with acetylation in PBMC Plasma VPA higher than in vitro effective concentrations	[44]

References are included at the end of the text

*A.I.* aromatase inhibitor, *5FU* 5-Fluorouracil, *5mC* 5-methyl Cytosine, *AUC* area under the curve (also a dosing calculation for Carboplatin), *Bid* bis in die (twice a day), *DLT* dose-limiting toxicity, *FEC* combination of Fluorouracil, Epirubicin, Cyclophosphamide, *FolFOx* combination chemotherapy of Folinic acid, 5-Fluorouracil and Oxaliplatin, *GI* gastrointestinal, *GIST* gastrointestinal stromal tumour, *HNC* head-and-neck carcinoma, *i.v.* intravenously, *MTD* maximum tolerated dose, *NSCLC* non-small cell lung cancer, *PBMC* peripheral blood mononuclear cells, *PD* pharmacodynamic, *PFS* progression-free survival, *PK* pharmacokinetics, p.o. per os (orally), *PR* partial response, *Pt* patient, *q* every (Latin “quaque”), *RA* rapid acetylator (Hydralazyne metabolism), *RD* recommended dose, *RR* response rate, *SA* slow acetylator (Hydralazyne metabolism), *SAHA* Vorinostat, Zolinza ®, *TS* Thymidylate Synthetase, target enzyme for 5FU activity, *VPA* Valproic Acid, *WBC* white blood cells

### Pharmacological aspects

An effective treatment, based on a sound scientific rationale, requires the identification of the relevant target(s) and the demonstration that the target(s) can be inhibited without excessive toxicity, and that the duration of this effect is sufficient to interfere with cell growth.

In the case of epigenetic treatment, it is necessary to prove that one of the different mechanisms that finely regulate gene expression (see above) is altered. Even if this mechanism is well established for many molecules, for some compounds such as CI-994 (N-acetyl-dinaline), an inhibitor of class I HDACs, the actual relevance of an epigenetic mode of action remains uncertain [27, 48, 49].

The list of epigenetic drugs is becoming longer every day and it also includes “old” drugs: Hydralazine, for example, has long been used as an anti-hypertensive agent, but it has recently been recognised as a demethylating agent [50, 51]. This also happens for HDAC inhibitors that include old molecules (such as valproic acid (VPA) [52] and a dozen compounds that have been recently synthesised [53].

#### Pharmacokinetics

To obtain an authentic effect on epigenetic mechanisms, the choice of the dose and schedule of administration is crucial since, especially for demethylating agents, if the drug concentration is too high this may result in cell toxicity or in a “traditional” antiproliferative effect. Furthermore, when a combination is used, the epigenetic effect must persist during chemotherapy (and possibly also later) in order to obtain an adequate synergism. This concept is guiding recent studies on new schedules of drug administration, as is the case for romidepsin [54].

Detailed data on pharmacokinetics have been published for several drugs (see Tables 1 and 2 for a complete list). The pharmacology of demethylating agents has been originally described in the past, and recent analyses mostly concern their interaction with other agents used in combination, such as temozolomide [55].

The pharmacology of HDAC inhibitors is particularly complex since many of these compounds act as enzyme inducers and may therefore modify their own kinetics when repeated dosing is used, or the pharmacology of associated drugs.

Drug concentrations in plasma are generally low, and a very sensitive assay, such as HPLC coupled to mass spectrometry, is required to obtain reliable data [56].

Individual characteristics may influence the kinetic parameters (pharmacogenetics): in the case of hydralazine, fast or slow metabolism is genetically determined, and in some studies, doses have been escalated or de-escalated according to individual metabolic parameters [57–59].

#### Pharmacodynamic effect

The most interesting part of the evaluation of anticancer drugs, particularly when dealing with an innovative mechanism of action, is the study of their effect in tumour cells. Several technical problems arise when trying to quantify the effect of epigenetic drugs, especially in solid tumours: it is difficult to decide what to measure, when and where.

Concerning demethylating agents, the effect is generally measured by evaluating global DNA methylation, but some authors determined the methylation status of specific genes that had previously been selected [60–62] and the level of expression of foetal haemoglobin has also been used as a PD marker [63]. The subject of DNA methylation may also be related to drug resistance caused by the activity of O(6)-methylguanine DNA methyltransferase (MGMT) that has been described in cerebral tumours [3, 64]. Decitabine has been used in an attempt to reduce methylation of genes involved in DNA repair in melanoma patients treated with temozolomide [55] but no such effect was evident even if decitabine caused hypomethylation of the HbF gene promoter.

The situation is more complex for HDAC inhibitors since not all drugs inhibit the different enzyme classes that are present in eukaryotic cells to the same extent. Some molecules have a wide inhibiting effect [65]; some are more restricted, and class-specific inhibitors such as CHR-3996, specific for class I HDACs enzymes [45], are being introduced in the clinics. Furthermore, the inhibition of de-acetylating enzymes may result in acetylation not only of histones, but also of other proteins, such as tubulin and Hsp90 that are involved in anticancer drug activity/resistance or in unrelated cellular pathways [66, 67].

An additional difficulty derives from the definition of a cut-off value: some authors, for example, required a doubling of histone acetylation to consider a result as “relevant” [68] but this was not mandatory in many other studies.

The effect of HDAC inhibitors was generally determined in terms of enzymatic activity, but in some cases histone acetylation, particularly H3 and H4, has been used as marker of activity. A consistent increase of H3 acetylation in peripheral blood mononuclear cells (PBMC) at effective doses has been observed with several agents, even if large inter-patient variations were often reported [69], and, more importantly, intratumoral H3 acetylation increase did not always correlate with response [70].

The effect of epigenetic treatment has also been evaluated by looking at specific genes in terms of expression [57, 62] or re-activation [71]. It is becoming clear that many elements are involved, and that it may be difficult to identify a consistent pattern in gene activation-inactivation [72] to be used as a marker of epigenetic activity.

The gold standard of pharmacodynamic studies is the evaluation of the effects in tumours. The study of malignant cells is certainly more complicated in patients with solid tumours than in leukaemia since cancer cells are more difficult to obtain, especially at multiple time points. Some reports on the evaluation of epigenetic therapy in solid tumour tissues have been published and deserve special consideration. The number of evaluable samples was often low, but informative results were obtained and reported [41, 73]. In ovarian cancer, it has been possible to evaluate the activity of demethylating agents on cells obtained from ascitic fluid and a gene-specific reduction of DNA methylation was evident [74]. Serial biopsies were obtained from patients with in head-and-neck carcinomas [70], glioblastoma [75], cervical and breast cancer and other tumours [57, 58, 66].

To overcome the difficulty in obtaining tumour samples, several groups have focused on the identification of surrogate markers. PBMC represent the most commonly used alternative. It is possible to measure HDAC activity and histone acetylation or DNA methylation. PBMC have also been used, in studies of drug combinations, to measure the target of non-epigenetic drug [76]. When PBMC and tumour biopsies were compared, however, results were not always consistent [66]. PBMC represent a “surrogate” tissue, and further improvement is required in order to make results obtained in these cells more representative of what is actually taking place in cancer.

A promising technique is the evaluation of circulating cell-free DNA [77] on which several specific analyses to identify epigenetic modifications can be performed [78]. The activity of demethylating agents has been evaluated by measuring the methylation status of circulating cell-free DNA in plasma in patients with refractory advanced non-small cell lung cancer (NSCLC) [62]. This is an interesting alternative with applications in many aspects of medicine, but further studies are required before it can be considered a reliable marker of epigenetic activity.

To obtain an adequate effect, sufficient drug concentrations must persist in the target cells for an adequate time. If there is a relationship between plasma kinetics and tissue effect (PK/PD relationship), drug dosing may be adapted on the basis of PK parameters that are easier to obtain. The permanence of a sufficient drug concentration or of a measurable effect has been evaluated in different tumour types and provides a rationale for the antitumour activity observed (see Tables 1 and 2 for details). Concerning the PK/PD relationship, data are difficult to interpret: no correspondence was found between plasma levels of valproate and histone acetylation in cervical cancer [79] so that PD assays may be required until we can devise more efficient PK models.

### Toxic side effects

One of the most interesting characteristics of many epigenetic drugs is that toxicity, at doses sufficient to achieve effective plasma concentrations, is generally very mild and has been described in detail in many studies of epigenetic agents used alone or in combination with standard anticancer agents (see Tables 1 and 2 for details).

#### Toxicity of demethylating agents

The best known agents that interfere with DNA methylation are decitabine and 5-azacytidine. More limited data are available for zebularine [46]. Since these agents also have traditional antiproliferative activity, the dose used plays a key role. At high doses, 5-azacytidine can cause neutropenia [68], similar to what is described for traditional anticancer drugs. It is generally assumed that in order to exploit the epigenetic action, it is necessary to use very low doses that are insufficient to cause any antiproliferative effect.

#### Toxicity of HDAC inhibitors

VPA is the best known molecule in this class since it has been used for many years as an antiepileptic drug: it is extremely well tolerated by patients, and also its long-term effects are well known. The most commonly reported complaints are neurological symptoms (such as dizziness) that are generally transitory and reversible [79, 80]. Neurological symptoms may become excessive when VPA is combined with other agents [63].

When vorinostat (SAHA) was tested in mice, relevant systemic toxicity was observed only at high doses [81]. In patients, anaemia, anorexia, hyperglycaemia, thrombocytopenia, fatigue and nausea have frequently been reported [82]. Similar toxic side effects were described for SB939 [83], for rosminostat [69] and romidepsin [70]. ECG abnormalities of different severity are the most concerning toxic side effects of CHR3996 [45] and of romidepsin [54].

### Antitumour activity

In a phase III trial of chemotherapy ± epigenetic treatment in cancer of the uterine cervix [59], there was an increase in progression-free survival, and the analysis of molecular correlates is pending.

Concerning phase II studies, that evaluated the antitumour activity of epigenetic agents alone or in combination with standard anticancer treatment, several studies have been reported. In many cases, results were described in terms of reduction of tumour volume in advanced disease resistant to several lines of anticancer treatments, similarly to what is standard for the evaluation of traditional antiproliferative agents [74, 82, 84, 85]. It is probably not surprising that several trials were reported as negative (see Tables 1 and 2 for details). Responses were evaluated according to Response Evaluation Criteria In Solid Tumors (RECIST): these may be useful for conventional anticancer therapies but do not seem adequate for epigenetic treatments that may result in disease stabilisation rather than in tumour shrinkage.

The evaluation of an epigenetic treatment, which is strictly connected to gene expression, can be performed more accurately in diseases where the genetic influence on the activity of the antitumour treatment is known. For this reason, the choice to evaluate the combination of entinostat and erlotinib in NSCLC was very sound [86]. The addition of the epigenetic agent, however, only improved progression-free survival (PFS) in tumours with high levels of e-cadherin, suggesting that this may represent a selection criterion for further studies.

The neo-adjuvant treatment of breast cancer represents a unique possibility in order to evaluate the activity of new agents. Not only new anticancer agents but also epigenetic therapy (hydralazine and valproate) have been tested in this setting [57], and the analysis of tumour biopsies confirmed that an epigenetic effect in terms of demethylation and of histone acetylation was detectable.

An interesting tactic was to evaluate the addition of epigenetic treatment in order to prevent or to reverse resistance due to the overexpression of a specific gene. This is the case, for example, of the increase in thymidylate synthase induced by fluorouracil treatment [87]. A clinical trial performed to demonstrate that this can be obtained in patients proved that treatment was feasible even if no clinically meaningful effect was obtained and no data were available for PD evaluation [88]. In a different study, patients with tumours progressing during standard chemotherapy were treated with the same regimen with the addition of hydralazine and valproate [58]. Data were analysed in detail, and even if tumour response was limited, there was evidence that adequate plasma concentrations were achieved and that an epigenetic effect was present. A similar approach was used in melanoma patients treated with temozolomide and decitabine: there was no antitumour activity even in the presence of a measurable PD effect [55].

## Conclusions

Epigenetic therapy is being more and more recognised as an effective and well-tolerated treatment of cancer. Data in leukaemias and myelodysplastic syndromes are now consistent, and the success obtained in cutaneous lymphomas represents a proof of principle that solid tumours may also respond. This is also supported by preclinical data; still clinical results fall short of expectations: several reasons may explain this discrepancy.

We are convinced that, similarly to what has been observed for tyrosine-kinase inhibitors in cancer [89], we need a better selection of tumours and of patients that may benefit from these treatments. It has already been stated that epigenetic drugs, and HDAC inhibitors in particular, “might be useful only in those tumours in which HDACs are directly involved in the pathogenesis” [27].

It is not surprising that clinical results have generally been disappointing: standard evaluation of anticancer activity, mostly based on tumour volume reduction, may not be an adequate index of activity for epigenetic treatment. Epigenetics is a complex mechanism of gene regulation: it will take time before we can exploit it at its best. We definitely need more appropriate tests to select potentially responding tumours, but we also need agents with demonstrated epigenetic activity and solid data in order to choose the most effective dose and schedule.

Several technical issues remain to be solved and this will keep researchers busy, both in preclinical and clinical settings, for a long time.

## Abbreviations

AdoMet: adenosyl-L-methionine
DNMT3-Like: DNA methyltransferase-3-like
DNMTs: DNA methyltransferases
FDA: Food and Drug Administration
H3: histone 3
H4: histone 4
HATs: histone acetyltransferases
HDACs: histone deacetylases
HMTs: histone methyltransferases
MDS: myelodysplastic syndromes
MGMT: O(6)-methylguanine DNA methyltransferase
ncRNAs: non-coding RNAs
NSCLC: non-small cell lung cancer
PBMC: peripheral blood mononuclear cells
PD: pharmacodynamics
PFS: progression-free survival
PK: pharmacokinetics
RECIST: Response Evaluation Criteria In Solid Tumors
SAHA: suberoylanilide hydroxamic acid (vorinostat)
VPA: valproic acid
